# Vertebral bone quality in patients with adolescent idiopathic scoliosis

**DOI:** 10.1007/s43390-026-01437-4

**Published:** 2026-05-22

**Authors:** Bernhard U. Hoehl, Luis Becker, Zhao Li, Zhen Wang, Hendrik Schmidt, Matthias Pumberger, Friederike Schömig

**Affiliations:** 1 Center for Musculoskeletal Surgery, Charité - Universitätsmedizin Berlin, Charitéplatz 1, 10117 Berlin, Germany; 2 Julius Wolff Institute, Berlin Institute of Health, Charité – Universitätsmedizin Berlin, Augustenburger Platz 1, 13353 Berlin, Germany

**Keywords:** Bone mineral density, Vertebral bone quality score, Adolescent idiopathic scoliosis

## Abstract

**Introduction:**

In adolescent idiopathic scoliosis (AIS), the choice of upper and lowest instrumented vertebrae and related complications remain debated. Bone quality is a relevant factor in this context. The vertebral bone quality (VBQ) score has recently been proposed as a radiation-free magnetic resonance imaging (MRI)-based measure of bone quality. However, the role of the VBQ score in AIS and its relationship with the severity of scoliosis characteristics has not yet been explored. We hypothesize that the VBQ score is associated with key features of AIS, including curve type, Cobb angle, lumbar modifier, and thoracic kyphosis.

**Methods:**

This retrospective study included AIS patients who underwent posterior spinal fusion between 2011 and 2025 at a university hospital. The lumbar VBQ score was determined using preoperative MRI, and the Lenke classification was assessed based on preoperative radiographs. To ensure comparability, curve types were categorized based on the flexibility of the lumbar curvature into non-structural (Lenke types 1 and 2) and structural (Lenke types 3 to 6) groups. The association between VBQ and curve type, Cobb angle, lumbar modifier, and thoracic kyphosis—while considering MRI field strength, age, sex, and body mass index (BMI)—was analyzed using multiple linear regression analyses.

**Results:**

This single-center study included 60 patients (male: 15%, female: 85%) with a median age of 15 years (interquartile range (IQR): 14–16 years) and a median BMI of 18.9 kg/m2 (IQR: 17.5–22.4 kg/m2), diagnosed with AIS presenting with non-structural (55%) and structural (45%) lumbar curves. A significant association was found with lumbar curve flexibility (r2 = 0.298, p = 0.035), indicating that structural lumbar curves correlated with lower bone quality. Additionally, female patients showed significantly better bone quality (r2 = -0.446, p = 0.005). No significant correlation was observed between the VBQ score and Cobb angle (r2 = -0.003, p = 0.370), thoracic kyphosis (r2 = 0.001, p = 0.734), lumbar modifier (r2 = -0.116, p = 0.123), MRI field strength (r2 = -0.181, p = 0.098), age (r2 = 0.018, p = 0.558), or BMI (r2 = -0.014, p = 0.275).

**Conclusion:**

Our study demonstrated a significant correlation between the VBQ score and both lumbar curve flexibility and sex.

Opportunistic assessment of the VBQ score may therefore be particularly useful in male patients with a structural lumbar curve to identify individuals who may benefit from further evaluation of bone quality.

## Introduction

Adolescent idiopathic scoliosis (AIS) is a common three-dimensional deformity of unknown origin that develops during adolescence in otherwise healthy children [1]. It is defined as a lateral curvature of the spine greater than 10° and vertebral rotation [2]. Surgery is considered for curves greater than 40° to 50° Cobb angle and depends on other factors including curve localization, curve progression, and skeletal maturity [3].

Despite its unknown origin, the reduced bone quality of patients with AIS was first described in 1982 [4]. Since then, reduced bone quality has been a constant topic of AIS linked to disadvantageous prognosis and complications: The degree of osteopenia correlates with curve progression and curve severity [5–7] and may prolong the duration of brace intervention while limiting the effect on the spinal curve [8, 9].

In AIS surgery, substantial variability exists among spine surgeons regarding both the number and placement of pedicle screws and the selection of instrumented vertebral levels. Although adverse outcomes related to level selection are uncommon, affected cases often require complex revision surgery. Therefore, improved preoperative planning is essential, as traditional “rules of thumb” increasingly fail to meet current standards of evidence-based practice [10].

These challenges are further exacerbated by reduced bone quality in AIS patients. The VBQ score has been shown to predict pedicle screw loosening [11]. In addition, osteopenia in AIS often persists and predisposes patients to osteoporosis, which not only amplifies the risk of implant-related complications but also increases the likelihood of fractures later in life [12–16].

In preparation for elective spine surgery, a multifactorial assessment is typically performed to minimize perioperative risks. However, in most patients, dual-energy X-ray absorptiometry (DEXA) is not routinely obtained prior to surgery [17, 18]. In the context of AIS, DEXA presents additional limitations: beyond its radiation exposure, the two-dimensional imaging may underestimate bone quality due to vertebral overlap in spinal deformities [19]. The magnitude of this underestimation has been reported at 11.5–17.5%, largely attributable to axial rotation and anteroposterior curvature [20].

In contrast, preoperative magnetic resonance imaging (MRI) is routinely performed in AIS patients to exclude anomalies such as tethered cord [21]. Building on this, Ehresman et al. introduced the Vertebral Bone Quality (VBQ) score, a simple measurement tool that quantifies bone quality based on fatty infiltration and trabecular atrophy in non-contrast T1-weighted lumbar sequences. A key advantage of this MRI-based approach lies in its three-dimensional assessment of spinal deformity, which enables more accurate measurements compared with DEXA [22] and provides a radiation-free method that could serve as a valuable screening tool for osteoporosis [23].

Previous studies have demonstrated clinical relevance of the VBQ score: higher values were associated with screw loosening and the need for reoperation after internal lumbar fixation [11, 24]. In AIS specifically, Ramos et al. reported significantly higher VBQ scores in affected patients compared with healthy controls, independent of Cobb angle [25]. Moreover, Wang et al. identified a significant correlation between a modified VBQ score of S1 and postoperative PJK in Lenke type 5 AIS patients [26].

Based on this evidence, we hypothesize that the lumbar VBQ score correlates with the morphological and functional characteristics of AIS—including lumbar curve flexibility, Cobb angle, lumbar modifier, and thoracic kyphosis—while accounting for sex, age, body mass index (BMI), and MRI field strength as potential confounding factors.

## Materials and methods

### Study design

This retrospective single-centre study received institutional ethical approval (EA2/216/22). We included patients with AIS who underwent spinal surgery between 2011 and 2025 at an academic medical centre. Indications for surgery included a Cobb angle > 40°, as well as additional factors such as curve localization and documented curve progression. Inclusion criteria comprised preoperative T1-weighted lumbar magnetic resonance imaging (MRI), standing full-spine radiographs, bending radiographs, and complete baseline demographic data, including age, sex, weight, and height. Exclusion criteria were age > 18 years, previous spinal surgery, and incomplete datasets precluding standardized measurements. The study was not restricted to a specific Risser stage.

We hypothesize that the VBQ score correlates with the characteristics of AIS (lumbar curve flexibility, Cobb angle, lumbar modifier, and thoracic kyphosis) when sex, age, BMI and MRI field strength are considered confounding factors.

### Variables

**Fig. 1:**
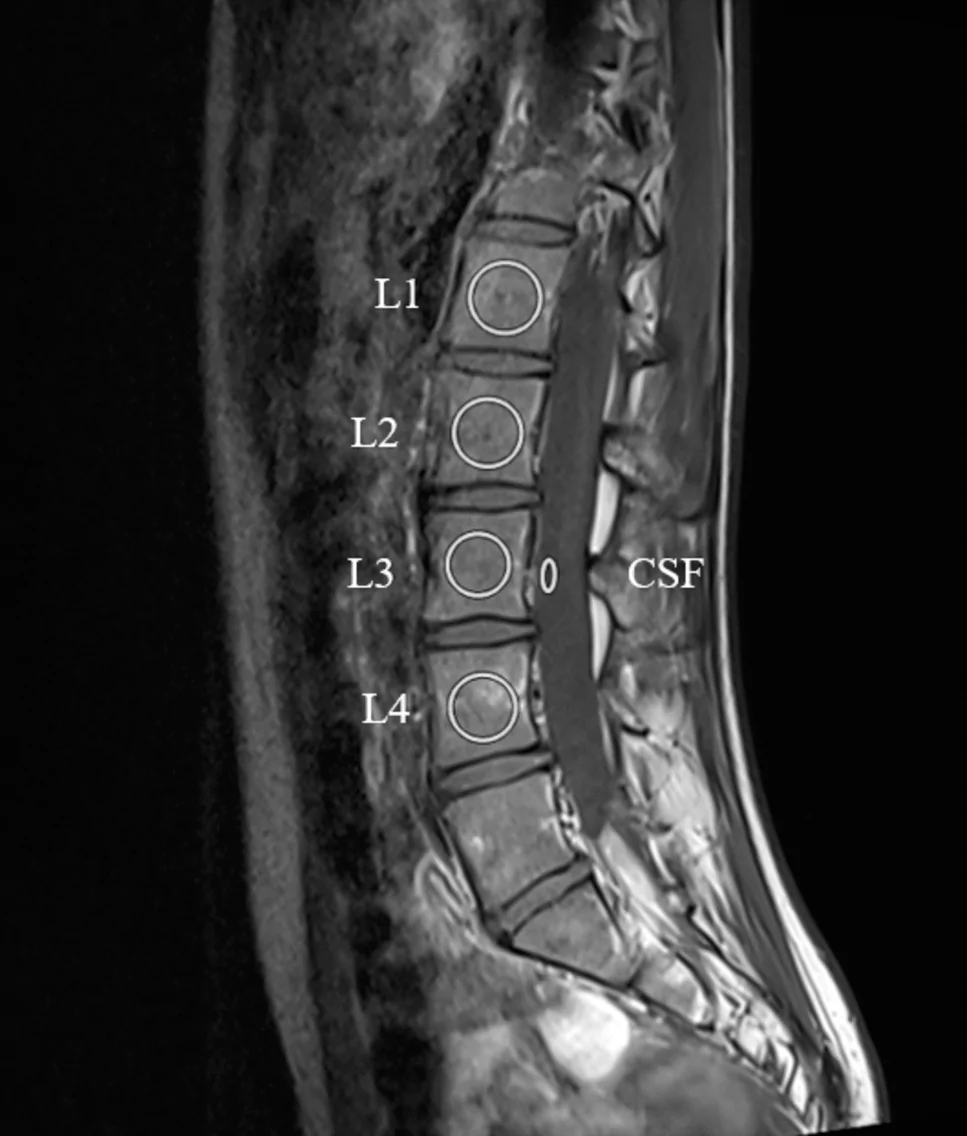
The vertebral bone quality (VBQ) score is measured in a mid-sagittal slice of a T1-weighted MRI. The median of the signal intensities of L1-L4 vertebral bodies are divided by the signal intensity of the cerebrospinal fluid (CSF) at the level of L3

The VBQ score was measured according to Ehresman et al. using mid-sagittal slices of a T1-weighted MRI of the lumbar spine [22]. The signal intensity of the trabecular bone within the L1–L4 vertebral bodies was measured by placing regions of interest over each vertebral body, (Fig. 1). To account for variability in signal intensity across different MRI scanners, the cerebrospinal fluid (CSF) signal at the level of the L3 vertebra was used as an internal reference. The VBQ score was calculated as the median signal intensity of the L1–L4 vertebral bodies divided by the CSF signal intensity. In cases of severe deformity, parasagittal slices were considered to guarantee the measurement of the signal intensity in the middle of the vertebral body. Cases with abnormalities preventing complete and correct measurements were excluded. Due to the impact of MRI field strength on the VBQ score, it was considered a confounding factor [27, 28]. For 1.5 Tesla (T), an Avanto MRI system (Siemens, Munich, Germany) was used with a repetition time (TR) of 522–950 ms, an echo time (TE) of 9.7–13 ms, and a slice thickness of 3 mm. For 3 T, a Magnetom MRI system (Siemens, Munich, Germany) was used with a TR of 589–862 ms, a TE of 9.2–10 ms, and a slice thickness of 3 mm.

All patients were classified according to the Lenke classification using standing radiographs. Structural curvatures were defined as persisting deformity over a 25° Cobb angle in bending radiographs. In cases with AIS with no persisting curvature of 25° Cobb angle in bending radiographs, the biggest curve was considered the single structural curvature. The variable Cobb angle was defined as the angle of the largest major curve. The lumbar spine modifier depends on the distance between the centre sacral line and the closest pedicle of the apex of the lumbar spine. Thoracic kyphosis was measured in the sagittal plane from the fifth to the twelfth thoracic vertebrae [29]. Based on the lumbar location of the VBQ measurement, curve types were classified in a binary manner according to lumbar flexibility, distinguishing non-structural curves (Lenke types 1 and 2) from structural curves (Lenke types 3–6).

All radiological data was measured using the Phönix-PACS software (Phönix-PACS GmbH, Freiburg im Breisgau, Germany). Age, sex, and BMI were collected from electronic medical records.

### Statistical analysis

Multilinear regression analysis was performed after collinearity diagnostic procedures to avoid multicollinearity among independent variables. The level of significance was set at *p* = 0.05. Collinearity was considered acceptable when the variance inflation factor (VIF) was below 5 [30]. Missing data were handled by complete case analysis to avoid bias due to imputation or estimation. Incomplete datasets were excluded prior to statistical analysis and documented. All statistical analyses were performed using R software (R Foundation for Statistical Computing, Vienna, Austria) [31] and RStudio software Version 2023.12.1.402 (Posit Software, PBC, Boston, MA, USA) [32]. The study was performed according to the Strengthening the Reporting of Observational Studies in Epidemiology (STROBE) statement [33].

## Results

### Participants

**Fig. 2:**
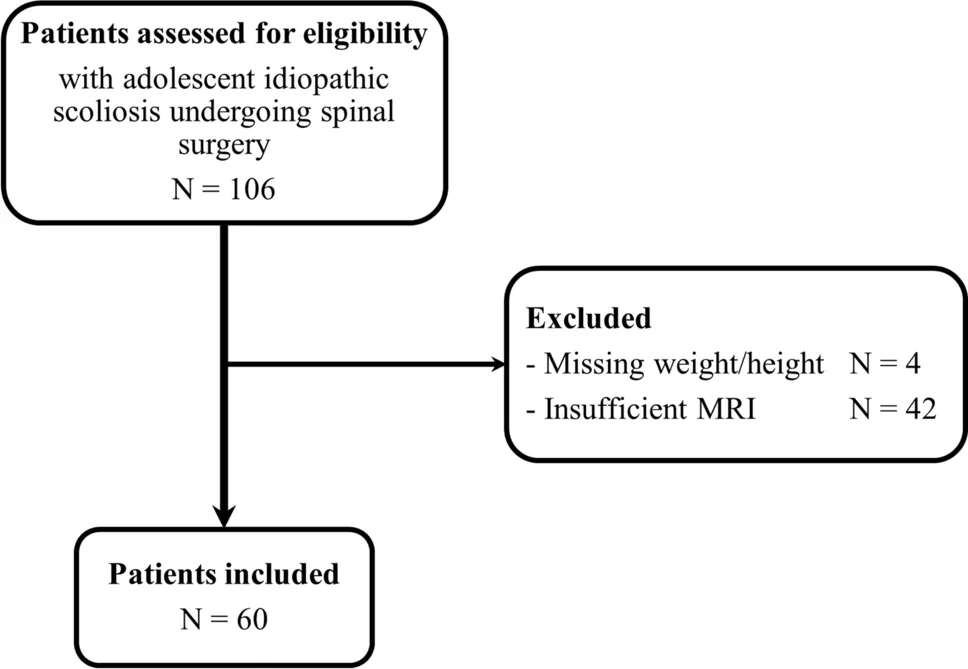
The Strobe diagram of the study

Between 02/2011 and 05/2025, 106 adolescent patients underwent primary spinal surgery for AIS. Fourty-two patients were excluded due to missing T1-weighted MRI of the lumbar spine. Four additional ones were excluded because basic information (weight and height) was missing (Fig. 2). Sixty patients with AIS met our inclusion criteria (Table 1).

**Table 1:** Patient characteristics are displayed separately for the groups split by lumbar curve flexibility (Non-structural lumbar curve (= Lenke 1&2) and structural lumbar curve (= Lenke 3&4&5&6))

Lumbar curve flexibility	Non-structural	Structural	Total
	*n* = 33 (55%)	*n* = 27 (45%)	*n* = 60 (100%)
Vertebral bone quality score	1.98 (1.76—2.16)	2.15 (1.83—2.29)	2.01 (1.81—2.25)
Male	2.42 (2.16—2.64)	2.43 (2.35—2.63)	2.43 (2.20—2.64)
Female	1.92 (1.71—2.07)	2.05 (1.81—2.26)	1.98 (1.78—2.16)
Field strength			
1.5 Tesla	12 (36%)	15 (56%)	27 (45%)
3 Tesla	21 (64%)	12 (44%)	33 (55%)
Sex			
Male	6 (18%)	3 (11%)	9 (15%)
Female	27 (82%)	24 (89%)	51 (85%)
Age [years]	15 (14—17)	14 (13—16)	15 (14—16)
Male	15 (15—16)	16 (16—17)	15 (15—16)
Female	15 (14—17)	14 (13—15)	15 (13—16)
Body Mass Index [kg/m^2^]	19 (17.4—21.6)	18.8 (17.8—23.6)	18.9 (17.5—22.4)
Male	18.5 (17.5—20.1)	22.5 (20.1—24.1)	19 (17.7—20.8)
Female	19 (17.5—22)	18.8 (17.9—23.3)	18.8 (17.5—22.5)
Risser stage	4 (3—4)	3 (3—4)	4 (3—4)
Male	3.5 (3—4)	4 (3.5—4.5)	4 (3—4)
Female	4 (3—4.5)	3 (2.5—4)	4 (3—4)
Lenke 1	26 (79%)		26 (43%)
Male	6 (23%)		6 (10%)
Female	20 (77%)		20 (33%)
Lenke 2	7 (21%)		7 (12%)
Male	0 (0%)		0 (0%)
Female	7 (100%)		7 (12%)
Lenke 3		8 (30%)	8 (13%)
Male		1 (13%)	1 (2%)
Female		7 (87%)	7 (12%)
Lenke 4		4 (15%)	4 (7%)
Male		1 (25%)	1 (2%)
Female		3 (75%)	3 (5%)
Lenke 5		6 (22%)	6 (10%)
Male		1 (17%)	1 (2%)
Female		5 (83%)	5 (8%)
Lenke 6		9 (33%)	9 (15%)
Male		0 (0%)	0 (0%)
Female		9 (100%)	9 (15%)
Cobb angle [°]	55 (47—68)	60 (51—72)	57 (49—71)
Male	54 (43—55)	60 (56—71)	54 (52—60)
Female	56 (48—70)	60 (51—72)	57 (50—72)
Lumbar modifier A	21 (64%)	4 (15%)	25 (42%)
Male	2 (10%)	0 (0%)	2 (3%)
Female	19 (90%)	4 (100%)	23 (38%)
Lumbar modifier B	7 (21%)	3 (11%)	10 (17%)
Male	3 (43%)	2 (67%)	5 (8%)
Female	4 (57%)	1 (33%)	5 (8%)
Lumbar modifier C	5 (15%)	20 (74%)	25 (42%)
Male	1 (20%)	1 (5%)	2 (3%)
Female	4 (80%)	19 (95%)	23 (38%)
Thoracic kyphosis [°]	28 (17—38)	24 (8—29)	25 (13—33)
Male	28 (11—41)	33 (29—61)	33 (18—42)
Female	28 (17—37)	23 (8—28)	25 (13—32)

Values are given by median (interquartile range). The percentual share of the corresponding category within the same subgroup is indicated in brackets

## Multiple linear regression

A multiple linear regression model was performed to control confounding field strength, sex, age, BMI, lumbar curve flexibility, Cobb angle, lumbar modifier, and thoracic kyphosis after collinearity diagnostic procedures (Table 2). Female patients showed significantly better bone quality (r2 = −0.446, p = 0.005). Additionally, a significant association was found with lumbar curve flexibility (r2 = 0.298, p = 0.035), indicating that structural lumbar curves correlated with lower bone quality.

**Table 2:** The multiple linear regression model was performed with field strength, sex, age, body mass index, lumbar curve flexibility, Cobb angle, lumbar modifier, and thoracic kyphosis as confounding factors

*Dependent variable:*						
VBQ score		Estimate		Standard error		*p*-value
Field strength (1.5Tesla/3Tesla)		−0.181		0.107		0.098
Sex (male/female)		−0.446^**^		0.151		0.005
Age at operation		0.018		0.031		0.558
Body mass index		−0.014		0.013		0.275
Structural lumbar curve (No/Yes)		0.298^*^		0.137		0.035
Cobb angle		−0.003		0.004		0.370
Lumbar modifier		−0.116		0.074		0.123
Thoracic kyphosis		0.001		0.003		0.734
Constant		2.795^**^		0.599		0.00003
Observations	60					
R^2^	0.313		Adjusted R^2^		0.205	
Residual Std. Error	0.390 (df = 51)		F Statistic		2.905^**^ (df = 8; 51)	
	^*^p < 0.05; ^**^p < 0.01					

*Sex (r*^*2*^ = *−0.446, p* = *0.005) and the lumbar curve flexibility (r*^*2*^ = *0.298, p* = *0.035) showed a significant correlation with vertebral bone quality (VBQ) scores*

## Discussion

In this study, the bone quality of patients with AIS was evaluated using the MRI-based VBQ score. A significant association was found with lumbar curve flexibility (r2 = 0.298, p = 0.035), indicating that structural lumbar curves correlated with lower bone quality. Additionally, female patients showed significantly better bone quality (r2 = −0.446, p = 0.005). There was no significant correlation of the VBQ score with Cobb angle (r2 = −0.003, p = 0.370), thoracic kyphosis (r2 = 0.001, p = 0.734), lumbar modifier (r2 = −0.116, p = 0.123), MRI field strength (r2 = −0.181, p = 0.098), age (r2 = 0.018, p = 0.558), or BMI (r2 = −0.014, p = 0.275).

To date, no studies have investigated whether the VBQ score correlates with different curve types or lumbar curve flexibility in AIS. Evidence from other diagnostic modalities assessing bone quality in relation to curve types is limited. Li et al. evaluated bone mineral density (BMD) using quantitative computed tomography and found no statistically significant difference between thoracic and lumbar curve types (p = 0.13) [34]. In contrast, Chen et al. demonstrated significant differences in femoral BMD when comparing Lenke type 1 (non-structural lumbar curve) and Lenke type 5 (structural lumbar curve) [35]. However, while the two-dimensional DEXA method remains the gold standard for BMD assessment [36], its accuracy is limited in patients with scoliosis with axial rotation exceeding 8° [37, 38]. Furthermore, various measurement methods also often exclude patients with severe lumbar deformities [39], making it challenging to reliably evaluate the relationship between lumbar curves and bone quality. Altered VBQ may influence load distribution and curve behavior during growth. Such potentially localized changes could contribute to progressive deformity and thereby promote the development of structural curves and specific curve patterns. However, this hypothesis remains speculative and requires confirmation in future studies.

Regarding sex differences in VBQ scores among adolescents, the available literature remains limited and heterogeneous. Patel et al. reported a mean sex-related difference in VBQ score of –0.155 (p = 0.035) in patients without AIS [40]. In contrast, two studies in adolescent AIS patients found no significant sex differences in VBQ scores: Yang et al. examined 136 AIS patients (mean age 14.84 ± 2.10 years) [41], while Wang et al. evaluated 206 AIS patients (mean age 14.4 ± 2.3 years) [42]. Notably, neither study stratified results by curve type.

Findings from BMD research are similarly inconsistent in adolescent patients with AIS. A recent systematic review and meta-analysis suggested that male AIS patients may have lower BMD and a higher prevalence of osteopenia, whereas earlier studies reported lower BMD in female patients [16]. Biological factors, such as differences in body size and the timing of pubertal onset, are known to influence these sex-related variations [43]. Approximately 90% of peak bone mass is attained by the end of the second decade of life, with nearly half of skeletal development occurring during adolescence [44]. On average, females tend to achieve higher BMD earlier, while males reach comparable levels only after the age of eighteen [45]. Subgroup analyses indicated that osteoporosis was significantly more prevalent among female patients, implying a less favorable prognosis, whereas male patients tended to present with osteopenia rather than manifest osteoporosis [16].

From a clinical perspective, these findings highlight the importance of considering both curve type and sex-specific bone development when evaluating patients with AIS. Although accurate assessment of bone quality remains challenging, particularly in cases of severe lumbar scoliosis, overlooking impaired bone quality may increase the risk of perioperative and long-term complications. Intraoperatively, reduced bone quality has been associated with increased blood loss [46] which may in turn contribute to perioperative metabolic or cardiovascular instability and elevate the risk of postoperative complications such as wound infections or hematomas [47]. Postoperatively, poor bone quality predisposes to mechanical complications, including a higher risk of screw loosening due to reduced pull-out strength [48] and an increased incidence of pseudarthrosis [49]. Moreover, Wang et al. reported that elevated VBQ scores were associated with an increased risk of PJK in AIS patients with Lenke type 1 and 2 curves (mean age 14.4 years) [42]. Beyond the perioperative setting, studies have shown that osteopenia in AIS persists and predisposes affected individuals to osteoporosis and an elevated risk of fragility fractures later in life [12–16].

The interaction between bone quality and spinal implants is strongly influenced by the screw selection and length of instrumentation. Given the complexity of spinal deformities and the multifactorial nature of complications, the selection of instrumented vertebrae remains a subject of ongoing debate [10]. Inappropriate selection of the upper instrumented vertebra may result in proximal anchor failure [50], increase the risk of PJK [51] or shoulder asymmetry reported in up to 25% of cases [52]. In addition, distal junctional kyphosis [53] and pedicle screw loosening [54] is also reported in the lower instrumented vertebra. While the choice of the lower instrumented vertebra determines the number of preserved lumbar motion segments, a more distal fixation may accelerate segmental degeneration [55] and contribute to low back pain [56]. On the other hand, distal curve progression may occur if fixation is insufficient, potentially resulting in loss of correction, coronal imbalance, and pelvic obliquity [57]. Such residual deformities can negatively affect body image, leading to chronic dissatisfaction, psychological distress, and reduced participation in daily activities and social interactions [55, 58].

Taken together, these considerations emphasize the clinical relevance of a comprehensive and multifactorial assessment of spinal deformity prior to surgical intervention. The VBQ score may provide additional value not only for surgical planning but also for long-term preventive strategies aimed at preserving spinal function and reducing postoperative complications.

This study has several limitations. First, it is a single-center retrospective investigation, which inherently limits the ability to assess longitudinal changes or establish causal relationships. Second, the study design carries a selection bias, as all included patients had severe AIS requiring surgical intervention. Consequently, the findings may not be generalizable to patients with milder forms of AIS. Due to the relatively small sample size in relation to the number of predictors, the risk of overfitting cannot be excluded: Although the multivariable regression model explains a moderate proportion of variance (R2 = 0.31) and the VIFs are below 2, the effect estimates should be interpreted as exploratory rather than definitive. Future comparative analyses involving less severe cases may help to reveal potential differences in bone quality across varying curve severities and lower Cobb angles.

## Conclusion

Our study demonstrated a significant correlation between the VBQ score and both lumbar curve flexibility and sex in patients with AIS. Given the vulnerability of this patient population, multiple factors must be considered to efficiently minimize long-term complications. A comprehensive, multifactorial assessment should integrate clinical examination, representative radiographs, and MRI to provide a reliable evaluation. Consideration of VBQ may enhance future decision-making processes for dorsal fixation, particularly in the selection of instrumented vertebral levels, and help reduce long-term complications.

## Data Availability

The data that support the findings of this study are available from the corresponding author, B.U.H., upon reasonable request.
